# Zinc Essentiality, Toxicity, and Its Bacterial Bioremediation: A Comprehensive Insight

**DOI:** 10.3389/fmicb.2022.900740

**Published:** 2022-05-31

**Authors:** Sarfraz Hussain, Maryam Khan, Taha Majid Mahmood Sheikh, Muhammad Zahid Mumtaz, Talha Ali Chohan, Saba Shamim, Yuhong Liu

**Affiliations:** ^1^Key Laboratory of Integrated Regulation and Resource Development on Shallow Lakes of Ministry of Education, College of Environment, Hohai University, Nanjing, China; ^2^Institute of Molecular Biology and Biotechnology, The University of Lahore, Lahore, Pakistan; ^3^Institute of Plant Protection, Jiangsu Academy of Agriculture Sciences, Nanjing, China

**Keywords:** zinc, bioremediation, pollution, wastewater, heavy metal, toxicity

## Abstract

Zinc (Zn) is one of the most abundantly found heavy metals in the Earth’s crust and is reported to be an essential trace metal required for the growth of living beings, with it being a cofactor of major proteins, and mediating the regulation of several immunomodulatory functions. However, its essentiality also runs parallel to its toxicity, which is induced through various anthropogenic sources, constant exposure to polluted sites, and other natural phenomena. The bioavailability of Zn is attributable to various vegetables, beef, and dairy products, which are a good source of Zn for safe consumption by humans. However, conditions of Zn toxicity can also occur through the overdosage of Zn supplements, which is increasing at an alarming rate attributing to lack of awareness. Though Zn toxicity in humans is a treatable and non-life-threatening condition, several symptoms cause distress to human activities and lifestyle, including fever, breathing difficulty, nausea, chest pain, and cough. In the environment, Zn is generally found in soil and water bodies, where it is introduced through the action of weathering, and release of industrial effluents, respectively. Excessive levels of Zn in these sources can alter soil and aquatic microbial diversity, and can thus affect the bioavailability and absorption of other metals as well. Several Gram-positive and -negative species, such as *Bacillus* sp., *Staphylococcus* sp., *Streptococcus* sp., and *Escherichia coli*, *Pseudomonas* sp., *Klebsiella* sp., and *Enterobacter* sp., respectively, have been reported to be promising agents of Zn bioremediation. This review intends to present an overview of Zn and its properties, uses, bioavailability, toxicity, as well as the major mechanisms involved in its bioremediation from polluted soil and wastewaters.

## Introduction

The surge in anthropogenic activities has exacerbated the problem of heavy metal pollution in the world, which, due to their indefinite persistence in the environment, are non-degradable pollutants and can be detrimental for living beings at high levels (Witkowska et al., 2021). Zinc (Zn) is one of the most profusely abundant transition elements in the Earth’s crust and is also reported to be an essential trace element for all living organisms (Roohani et al., 2013; Jarosz et al., 2017; Lee, 2018). It is a significant component of various proteins, acting as a cofactor or coenzyme of more than 300 enzymes (Rahman and Karim, 2018), and is attributable to the activity and regulation of various enzymes, proteins, DNA and DNA binding proteins, immunity, as well as cell metabolism. It also aids in suppressing the generation of free radicals and reactive oxygen species (ROS), which accentuates protein stability and the function of antioxidative enzymes (Narayanan et al., 2020). In nature, it exists as a divalent cation (Zn^2+^), thus interacting with various negatively charged ions (CO_3_^−^, OH^−^, C_2_O_4_^2−^). Zn was first reported to be essential for the growth of microorganisms in 1869 (Raulin, 1869), much earlier than plants and animals (Todd et al., 1933; Prasad et al., 1963). It was not until the 1960s that Zn was established to be essential for humans as well, prior to which it was not known that its deficiency would cause detrimental effects to human health (Prasad, 2017). In humans, Zn serves the elemental role in the production of hormones and their receptors (Chasapis et al., 2012; Roohani et al., 2013). It is reported that the cumulative concentration of Zn in the human body amounts to approximately 2–3 gm, more than 80% of which is apportioned in the bone and muscles. During transportation within the body, enzymes like superoxide dismutase and carbonic anhydrase in the red blood cells carry the largest quantity of Zn in the bloodstream (Vallee and Falchuk, 1993). However, majority of transported Zn is bound to albumin in plasma, with approximate concentration of 12–16 μm (Rink and Gabriel, 2000). Furthermore, the regulation of Zn in the human body is closely associated with normal levels of cellular proliferation, apoptosis and differentiation (Haase and Rink, 2014a), and the production of cytokines and antibodies, thus serving a significant role in the homeostasis of innate and adaptive immune systems (Haase and Rink, 2014b; Weyh et al., 2022). The supplementation of Zn has also been recommended for the reduction of viral activity, as well as the amelioration of tissue damage and inflammation in many viral infections, including COVID-19 (Asl et al., 2021; Rodelo et al., 2022). The consumption of Zn in humans is typically dependent upon dietary intake, which is varied according to age and gender. According to the German Nutrition Society, the consumption of 1.5 mg/day for infants (0–4 months), 11 and 14 mg/day for adolescent males and females (15–19 years) has been advised, while for adult males and females (ages 19 or older), the average allowance of intake is recommended to be 11–16 and 7–10 mg/day (variable by phytate intake), respectively, by The Food and Nutrition Board, United States (Otten et al., 2006; Rodelo et al., 2022). Thus, deficiency of Zn can be an important factor in creating nutritional imbalance and can adversely impact human health, where it can be a precursor in the development of certain medical ailments, including immune dysregulation, impaired immunity, increased vulnerability to infection, lymphopenia, common cold, epilepsy, acne, Wilson’s disease, and neurological diseases, such as schizophrenia, Parkinson’s, and Alzheimer’s disease (Irving et al., 2003; Walker and Black, 2004; Prasad, 2013). Zn intake and its excess is also reported to be associated with the modulation of gastrointestinal flora and immune systems (Skalny et al., 2021). In this light, it is also important to note that excessive intake of this element can have negative effects, such as disturbances in the levels of other elements, particularly copper, which can subsequently result in copper deficiency anemia, reduction in the copper-dependent enzymes, and cholesterol metabolism dysregulation (Terrin et al., 2020; Brzóska et al., 2021).

In literature, there are various reports which demonstrate the adverse effects caused by excessive micronutrient exposure, including exposure to Zn, causing disturbance to the homeostasis of various biological systems (Hynninen et al., 2010; Potocki et al., 2012). At higher concentrations, Zn can cause toxicity in cells, which can often result in the disruption of essential biological functions triggered by blocking protein thiols through mismetallation with other metals (Marchetti, 2013; Chandrangsu et al., 2017). In the environment, emissions of vast quantities of Zn due to natural and anthropogenic sources has enabled its unmitigated entry into food chains, resulting in biomagnification in living organisms. Bioremediation is feasible and efficient method for the removal of these toxic quantities from contaminated soil and wastewater sources, thus offering a new perspective on the utilization of heavy metal-resistant bacteria through sustainable development (Sharma et al., 2021).

This review aims to compile and present the chemical and physical properties of Zn, its uses, the effects of its toxicity, as well as summarize and highlight the potential mechanism of resistance in bacteria which enable them as promising agents of bioremediation of heavy metals, such as Zn.

## Physical and Chemical Properties of Zinc

Zinc is represented by the symbol “Zn,” with an atomic number and weight of 30 and 65.38 gm, respectively. It was discovered and recognized as a metal for the first time in 1746 by German chemist Andreas Sigismund Marggraf (National Center for Biotechnology Information, 2022). It belongs to group XII (known formerly as II-B) of the periodic table (Period number 4, element group number 12, block “d”; Jensen, 2003), and is a brittle, lustrous, bluish-white metal which is solid at room temperature. When heated at temperatures above 110°C, it readily becomes malleable and ductile and is generally considered to be a moderately reactive metal in terms of its reactivity with oxygen and other metals (Table 1; Wuana and Okieimen, 2011). It acts as a strong reducing agent, and in hydrolytic reactions, acts as a Lewis acid in catalyzing reactions, thereby being associated with various metalloenzymes, DNA binding, and regulatory proteins, as well as transcription factors (Cerasi et al., 2013). Zn naturally occurs as a mineral sphalerite (ZnS) and has five stable isotopes (^64^Zn, ^66^Zn, ^67^Zn, ^68^Zn, and ^70^Zn) in nature, from which ^64^Zn is the major isotope in terms of natural abundance (Audi et al., 2017).

**Table 1:** Description of various physical parameters of Zn.

Physical parameters	Zn properties
Atomic number	30
Density at room temperature (gcm^−3^)	7.134
Atomic weight (mass)	65.39
Relative abundance in Earth’s crust (%)	8 × 10^−3^
Melting point (°C)	419.53°C
Boiling point (°C)	907°C
Heat of vaporization (kJ/mol)	295.8
Specific heat capacity (J kg^−1^ K^−1^)	388
Heat of transformation, (J/gram atom)	1,966
Crystal structure; α-Zn β-Zn	Cubic (face-centered) Tetragonal
Van der Waals radius (nm)	0.138
Ionic radius (nm)	0.074
Isotopes	5 (stable)
Electronic shell	[Ar] 3d^10^ 4s^2^
Energy of first ionization (kJ mol^−1^)	904.5
Energy of second ionization (kJ mol^−1^)	1723
Energy of third ionization (kJ mol^−1^)	3832.687

Zn does not generally act as a typical metal, as it does not have an 8-electron octet rather an 18-electron shell upon the loss of its outermost two electrons, rendering it less reactive than other metals. However, since it undergoes neither oxidation nor reduction, it forms a stable metal ion in biological matrices where the redox potential is in constant flux (Graf, 1997). It has an oxidation state of 2+, and readily forms compounds with ammonia, cyanide, and halide ions. When compared to the metal itself, Zn dust is reported to be more reactive and pyro-phoric due to its large surface area. Selective compounds of Zn (chloride and sulfate) are water soluble, whereas other compounds (sulfide, carbonate, phosphate, oxide) are observed to be insoluble or slightly soluble in water (Habashi, 2013). It is capable of displacement of all metals beneath its position in the electrochemical series, and can also displace gold from cyanide solution, the latter of which is a large-scale industrial process (Robinson and Pohl, 2013). The surface of the metal is prone to rapid corrosion, ultimately forming an encapsulant layer of Zn carbonate after reacting with atmospheric carbon dioxide (Holleman et al., 1985), though it can be removed by the corroding action of strong acids (HCl, H_2_SO_4_; Porter, 1994).

## Immunomodulatory Functions of Zinc

### Zinc and Its Role in Innate and Adaptive Immunity

Sufficient consumption and uptake of Zn is vital for the regulation and optimal function of innate and adaptive immunity in human beings (Bonaventura et al., 2015). In innate immunity, Zn serves an elemental role in maintaining the activity of NADPH oxidase found in neutrophil granulocytes (Hasegawa et al., 2000; DeCoursey et al., 2003). Thus, the lack of Zn uptake and its subsequent deficiency could beget decreased production and killing potential of ROS (Bonaventura et al., 2015). Furthermore, Zn deficiency mediates the induction of decreased adhesion and chemotactic behavior in neutrophil and monocyte granulocytes and impairs the function and maturation phase of macrophages (Shankar and Prasad, 1998). Pertaining to its role in natural killer cells (NK cells), a deficit of Zn in the body could reduce overall NK cell count in blood, where the decreased chemotactic and lytic behavior of infected or cancer cells has also been observed (Rajagopalan et al., 1995; Rajagopalan and Long, 1998).

In the case of adaptive immunity, the presence of Zn is fundamental in the generation, maturation, and activity of T cells (Fraker and King, 2004). This is due to its significance in the elemental composition of thymulin, a hormone produced in the thymus which promotes the development of pre-T lymphocytes into mature lymphocytes (Dardenne and Pleau, 1994; Saha et al., 1995), which can be inhibited in the case of Zn deficiency, resulting in thymus atrophy and decreased T-cell count (King et al., 2005). Moreover, Zn deficiency can negatively affect T cell and cytokines (such as IL-2 and IFN-*γ*) production (Bădulici et al., 1994; Prasad et al., 1997). Apart from T-cell maturation, Zn also aids in the cell differentiation process, where it was observed that a deficiency of Zn demonstrated a decrease in CD4^+^ T cells, which ultimately results in the disruption of CD4^+^/CD8^+^ cells ratio, which is a characteristic sign of immune dysfunction (Beck et al., 1997; Sheikh et al., 2010).

### Immunomodulatory Effects of Zinc

Apart from its effect on the functions of selective immune cells, the uptake of Zn is affiliated with the general regulation of the entire immune system, where various studies have demonstrated that elevated levels of oxidative stress and inflammation are all associated with a deficiency of Zn in the body (Wong et al., 2015, 2019; Gammoh and Rink, 2017). In *in vivo* models, the supplementation of Zn was observed to induce the production, maturation, and stability of function in regulatory T cells (Rosenkranz et al., 2016, 2017). Moreover, lack of Zn in the body can aggravate cases of chronic inflammation, which can elevate the expression of pro-inflammatory markers, such as IL-1*α*, IL-1β, and IL-6 in several inflammatory disease (Yazar et al., 2005). This aspect of Zn significance as a micronutrient is therefore essential for the positive downregulation of disease pathogenesis and molecular pathways, such as NF-κB signaling pathway, which significantly regulates apoptosis, immune and inflammatory responses, as well as the expression of pro-inflammatory cytokines (Jarosz et al., 2017). In addition, Zn also importantly affects the expression of Zn finger proteins which are known to suppress the expression of TNFR- and TLR-initiated NF-κB signaling pathways (Gammoh and Rink, 2017).

### Zinc and Infections

Over the years, studies have reported the importance of Zn in the alleviation of viral infections, which is mainly associated with the entry of virus particles into the host cells, and their subsequent fusion and replication, as well as translation and emission of virus proteins in the host (Ishida, 2019; Read et al., 2019). In a study, it was reported that supplementing Zn at the recommended dose (>75 mg/day) remarkably aided in the reduction of severity of colds (Maares and Haase, 2020). In elder adults, the supplementation of Zn (45 mg/day of Zn gluconate) was observed to reduce the number of infections, as well as strengthen their immune system *via* the marked increase in the concentration of Zn in the plasma, along with the lowered production of oxidative stress markers and TNF-α (Prasad et al., 2007). More recent research findings demonstrate the ability of Zn to aid in the inhibition of RNA polymerase of SARS virus, *via* the reduction of viral replication (Te Velthuis et al., 2010), which are suggestive of Zn to be potentially effective against SARS-CoV-2, and COVID-19 (Skalny et al., 2020; Weyh et al., 2022).

## Bioavailability of Zinc

Plants tend to uptake Zn from soil because of which its deficiency is reported to be a significant abiotic stress factor affecting more than 40% of agricultural lands worldwide. Several plant species are reported to be Zn-efficient (rich in Zn), such as carrot, rye, cashew nuts, wheat, sunflower, pea, oats, and alfalfa (Hambidge et al., 2010). Other good sources of Zn include beef, and dairy products, such as milk, eggs, and cheese (Hacisalihoglu, 2020). Though it is well established that the presence of high phytate ions in the diet can ultimately hinder the absorption of several essential minerals like Ca, Fe, and Zn (Castro-Alba et al., 2019), improved absorption of Zn was observed when dairy products were consumed in combination with other foods with high phytate (e.g., bread, tortillas, and rice), which seemed to be associated with the presence and beneficial effects of citrate and phosphopeptides in dairy products (Shkembi and Huppertz, 2021). Zn absorption occurs within the small intestine in its ionized form which is formed when Zn is released from consumed foods. However, this ionized form may then be able to form inhibiting complexes with ligands of organic acids, such as phytates, amino acids, and phosphates, inadvertently affecting its solubility and absorption (Krebs, 2000). The presence of citrate, a low molecular weight ligand found in human milk which binds with Zn, and casein phosphopeptides, which are phosphorylated peptides released upon the digestion of casein which can also bind to Zn, in dairy products has thus been hypothesized to improve Zn absorption by preventing it from binding and forming complexes with phytate ions (Miquel and Farré, 2007). However, there have been some reports which state otherwise (Pécoud et al., 1975; Sandström and Cederblad, 1980; Flanagan et al., 1985; Wood and Zheng, 1990). The availability of Zn from mixed- or vegetable-based diets is reported to be more than 20% (Hacisalihoglu and Blair, 2020). The use of Zn supplements is widespread, though not necessarily mandatory for fulfilling dietary Zn requirements. In a normal and healthy host, the choice of Zn salt used is insignificant (Tran et al., 2004), though the amount of Zn absorbed from supplements is more greater than absorption from diet. Furthermore, despite the beneficial effect of Zn consumption on the immune system, excessive intake can result in its toxicity (Maares and Haase, 2020).

## Zinc Toxicity

Heavy metals are known to persist in the human body as well as the environment, with various environmental sources being attributable for their wide availability (Haiyan and Stuanes, 2003; Nadal et al., 2005). They can usually be exposed to humans through inhalation, ingestion, and dermal contact (of contaminated soil and water), as well as consumption of contaminated produce (Hough et al., 2004; Baastrup et al., 2008; Mari et al., 2009; Man et al., 2020). In order to ensure public safety and health, it is essential to identify and mitigate these sources of exposure (Hu and Dong, 2011). Zn is one of the most readily available heavy metals found in soil, where its excessive levels may exert a phytotoxic effect which can drastically affect crop quality and yield, respectively, and even pose a health risk to humans upon consumption due to the accumulation of Zn through absorption or deposition (Wang et al., 2005; Baran, 2012, 2013; Bolan et al., 2014). Furthermore, excess levels of Zn in soil also contribute to the inhibition and alteration of soil microorganisms (Olaniran et al., 2013), due to its bioavailability in the form of ionic Zn and as part of organo-metallic complexes, which have been previously determined (Pueyo et al., 2004; Anju and Banerjee, 2011; Fedotov et al., 2012; Agnieszka et al., 2014; Kim et al., 2015; Baran et al., 2018). Therefore, efficient methods of heavy metal remediation are crucial for the alleviation of Zn toxicity in order to preserve soil microorganisms and their biodiversity, as well as plant and human health (Guarino et al., 2020).

Zn is a biologically essential element for the human body, which is significantly required for the normal function and regulation of several enzymes and other proteins. However, excess levels of Zn can act as toxic, which can induce conditions of acute toxicity for living beings (Lindholmer, 1974; Wu et al., 2011; Wadige et al., 2014). According to the Environmental Protection Agency, United States, the Zn toxicity concentration in the case of freshwater aquatic life is 120 μg/l (for short- and long-term hazardous concentration), while the ranges for human health amount to 7,400 and 26,000 μg/l, respectively (for drinking water + consumable aquatic living beings, and consumable living beings only; US EPA, 2009; Li et al., 2019). In humans, Zn toxicity mainly occurs in either of three ways; oral, dermal, and inadvertent inhalation (Toxicological Profile for Zinc, 2005). As most of Zn toxicity cases are reported to be acute, prognosis are often good with complete recovery by treatment, such as chelation therapy or medication (Qu et al., 2012). One of the most common causes of acute Zn toxicity is the over-consumption of dietary Zn supplements. This also creates an imbalance for copper bioavailability, which can subsequently lead to its deficiency. This mechanism can likely be attributable to Zn-induced formation of copper-binding metallothioneins (Sandstead, 2013).

**Figure 1:**
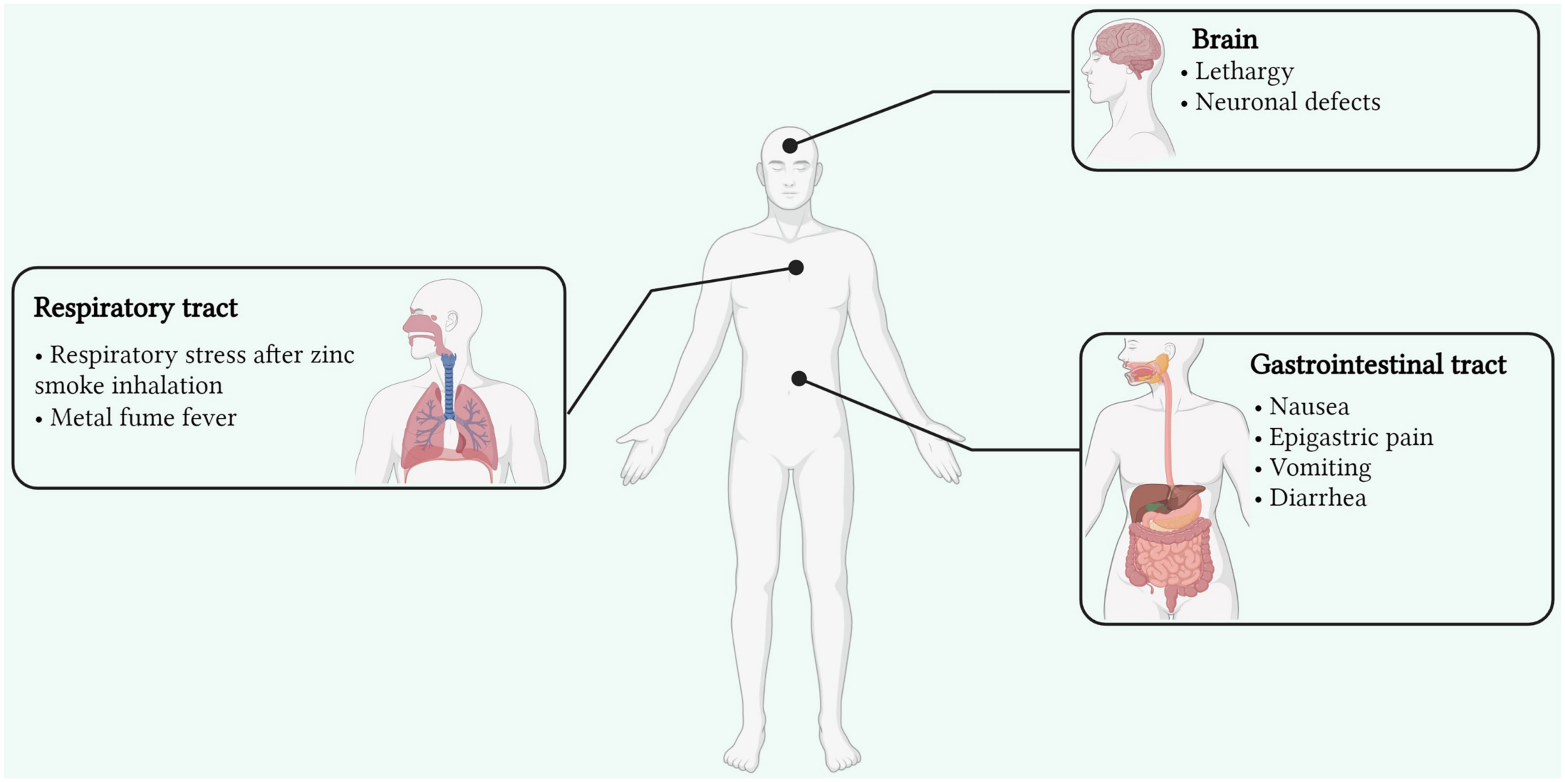
Signs and symptoms of Zn toxicity affecting various organs and sites of the human body (Created with BioRender). Zn toxicity can occur *via* three primary routes; oral, dermal, and inadvertent inhalation. Though reversible, its toxicity can affect the respiratory and gastrointestinal tracts as well as the brain with various side effects. Inhalation of Zn fumes can induce metal fume fever arising from fume inhalation in industries, manifesting several symptoms in the case of acute exposure which include nausea, muscle fatigue, chest pain, cough, breathing distress, and fever.

Other reasons include the unpremeditated consumption of dental products rich in Zn, as well as Zn contaminated food and drinks, which can result in acute gastrointestinal illness, with vomiting, nausea, and epigastric pain as the major symptoms. Inhalation of smoke and fumes containing Zn usually occurs in the case of exposure to industrial processes, such as galvanization. Furthermore, Zn-containing smoke bombs are the major sources of inhalation toxicity, primarily in soldiers and armed forces (Freitag and Caduff, 1996). However, in two reports, there was no scientific evidence that the respiratory distress was mainly attributable to Zn (Johnson and Stonehill, 1961; Zerahn et al., 1999). Inhalation of Zn fumes can induce metal fume fever, which is one of its most widely caused and reported effects (Figure 1). It is an acute condition originating in cases of industrial exposure (Zn smelting, welding, and galvanization) in the presence of metal fumes (particular size <1 μm; Vogelmeier et al., 1987). Although a non-life-threatening condition, it can manifest several symptoms in the case of acute exposure which include nausea, muscle fatigue, chest pain, cough, breathing distress, and fever (Rohrs, 1957; Putila and Guo, 2011). With accurate treatment, these symptoms can be cured completely within a matter of days (Martin et al., 1999; Plum et al., 2010). Excessive consumption of Zn can also result in gastrointestinal distress, with several symptoms, such as vomiting, nausea, cramps, diarrhea, and epigastric pain. This can also arise from ingesting Zn from food and drinks stored in galvanized containers, where the acidic nature of the food or drinks enables the liberation of Zn from the galvanized coating layer (Brown et al., 1964). Ingestion of Zn sulphate tablets was also reported to induce gastrointestinal stress symptoms in healthy subjects (Haase et al., 2008). Additionally, other compounds, such as Zn gluconate and oxide, are also observed to induce similar effects on human gastrointestinal health (Callender and Gentzkow, 1937; Lewis and Kokan, 1998; Liu et al., 2006). In human health, though Zn is well-reported to be essential for the regulation of most biological functions, its excessive levels are implicated in the causation and development of prostate cancer (Zaichick et al., 1997; Costello and Franklin, 1998), which is associated with regulating Zip1, a Zn transporter attributed to its accumulation and acquisition in prostate cells (Costello et al., 1999; Franklin et al., 2005). Though men having a moderate to high level of Zn intake may be reported to face a lower risk of prostate cancer, exceedingly high concentrations or perpetual Zn intake can result otherwise (Jarrard, 2005; Plum et al., 2010).

## Zinc in the Environment

In the environment, the persistence of Zn is the culmination of both natural and anthropogenic sources, the latter of which can arise from various sources (Hanahan and Weinberg, 2000). These sources can significantly contaminate water sources when industrial waste is discharged into river streams and water lines, where microbial and marine life get exposed. Industrial wastewaters can also be released into soils and irrigative lands where this contaminated water can enable Zn to leach into soils and affect soil microbial diversity and pH (Gautam et al., 2016).

Zn is the one of the most abundant elements (23rd in rank) found in the Earth’s crust, with an average of about 78 mg/kg (Alloway, 2008). It is deemed requisite for modern times, as it is one of most utilized metals alongside iron (Fe), aluminum (Al), and copper (Cu) in the world in terms of tonnage. Sphalerite, Zn’s primary ore, is among the most principal ores in the world. Zn mine global production was reported to increase in 2021 from that of its predecessor year, where production was observed to be halted due to lockdown restrictions (International Lead and Zinc Study Group, 2020). Therefore, in 2021, production reached approximately 14.13 million tons which resulted in a production surplus (Guo and Feng, 2013). The identified Zn resources are reported to amount to more than 1.8 billion tons in the world, with China being the second largest country in the world in terms of extracting, importing, and consumption of Zn (Li et al., 2019; U.S. Geological Survey, 2022). The natural introduction of Zn in soils is attributable to natural phenomenon of parent rock erosion and weathering. The exchangeability and bioavailability of Zn (in Zn^2+^ form) is dependent upon the pH of the soil, where adsorption and desorption into soil organic matter takes place (Mertens and Smolders, 2013). However, at high concentration of Zn, non-specific sorption to clay as well as mineral precipitation are primary and there is little or no correlation to pH. Typical concentrations of Zn in the soil range from 10–300 mg kg^−1^ (White, 1993). Zn concentration is reported to be richer in soils formed from basic rocks (e.g., basalt) as compared to acid rocks (e.g., granite; Vinogradov, 1959). Furthermore, Zn content is found to be comparatively greater in heavier soils than lighter ones (Frank et al., 1976). Other sources may likely include atmospheric deposition through natural sources like volcanic ash, forest fires, and dust (Nriagu, 1989), along with man-made sources, such as combustion of fossil fuels, galvanization, tire and railing rust, motor oil, cement, tar production, and hydraulic fluid (Imseng et al., 2019). In soil, Zn can occur in either of these five forms; water soluble, complexed, chelated, adsorbed, or exchangeable; where these forms can vary in terms of uptake, dissemination, and strength and are regulated by several factors, such as pH, and the concentration of other ions, such as Fe and manganese (Mn; Deb, 1992). Moreover, the availability of Zn to plants in the soil is contingent on clay, carbonate, and total Zn content, redox, and moisture conditions, microbial activity in soil, as well as the concentration of micro- and macro-nutrients (Mandal et al., 1993; Alloway, 2008). In soils formed from limestone, the chemisorption of Zn onto calcium carbonate aids the formation of Zn hydroxycarbonate which ultimately inhibits its availability to surrounding plants. Moreover, Zn readily adsorbs to kaolinite and illite types of clay in the presence of high pH conditions (Farrah and Pickering, 1977). The formation of poorly soluble Zn sulfide is facilitated by the oxidation of organic matter in the case of Zn sedimentation, which also decreases the availability of Zn to plants (Sandstead, 2013). In the atmosphere, Zn is usually found in its oxidized form, and particles containing Zn can range up to 5 mm in size. In freshwaters, the pH range most often favors the adsorption of Zn, where its binding to organic matter is facilitated by pH above 6. Normal concentration of Zn in surface and groundwater may be <10 μg/l and about 10–40 μg/l (Elinder, 1986; Sandstead, 2013). Industrial uses of Zn span a wide range of applications, including galvanization, production of brass, bronze, Zn-based alloys, metal-coating, paint, dyes, and pigments (Incharoensakdi and Kitjaharn, 2002; Wu et al., 2017).

## Zinc Resistance Mechanisms and Bioremediation

The worldwide dilemma of environmental pollution has been aggravated by the incessant usage of heavy metals in various industries. According to the US EPA, more than 40% of industrial wastewaters are reported to be contaminated with heavy metals and organic pollutants (Sharma et al., 2018). To tackle this worsening situation, many physical, chemical, and biological methods have been introduced over the years to successfully remove these pollutants from wastewater. Physical and chemical methods have since been proven to be exorbitant and unfeasible at large-scale applications (Wang and Chen, 2009). In this regard, developing and promoting biological methods to alleviate toxic levels of heavy metals was imperative. Therefore, the introduction of novel approaches has unlocked the usage of biological sources (microorganisms, plants as well as algae) to combat this problem (Ahemad and Malik, 2012). Plants have evolved to adapt numerous defense mechanisms against the exposure of excess heavy metals, including complexation, chelation with metallothioneins, and migration with ligands *via* plasma membrane channels (Cunningham et al., 1995; Wei et al., 2021). This mechanism is known as phytoremediation. Furthermore, bacteria, yeast, fungi, protozoa, and algae that grow despite the harsh environment of industrial wastewaters have evolved to harbor various resistance mechanisms that can aid in their survival (Zahoor and Rehman, 2009). Bioremediation is the process which employs the use of biological agents for the removal of heavy metals from various environments, particularly effluents and wastewaters, which has since been hailed as a cheaper, more efficient method than chemical and physical ones (Timková et al., 2018). As the problem of pollution is getting more pronounced all over the world, more and more importance is being given to detoxification methods of effluents prior to their release (Shamim and Rehman, 2012). In soil, excessive levels of heavy metals may induce alteration to soil microbial diversity and selective survival in soil microbes (Berendonk et al., 2015; Epelde et al., 2015). This can be mitigated by adapting an efficient strategy for the evolution of microbes, based on encoded genetic mechanisms, a phenomenon found in several bacterial strains which survive in heavy metal-polluted areas (Aka and Babalola, 2017). Therefore, bacteria can be utilized for remediating heavy metals from polluted areas (Chen et al., 2018). Though many efforts regarding heavy metal resistance mechanisms in bacteria are majorly focusing upon single isolated strains, a comprehensive elucidation of resistance mechanisms on the whole, contaminated areas, and the feasibility of such strategies are crucial for the optimization of bioremediation (Chen et al., 2019).

### Mechanisms of Zinc Homeostasis in Bacteria

**Figure 2:**
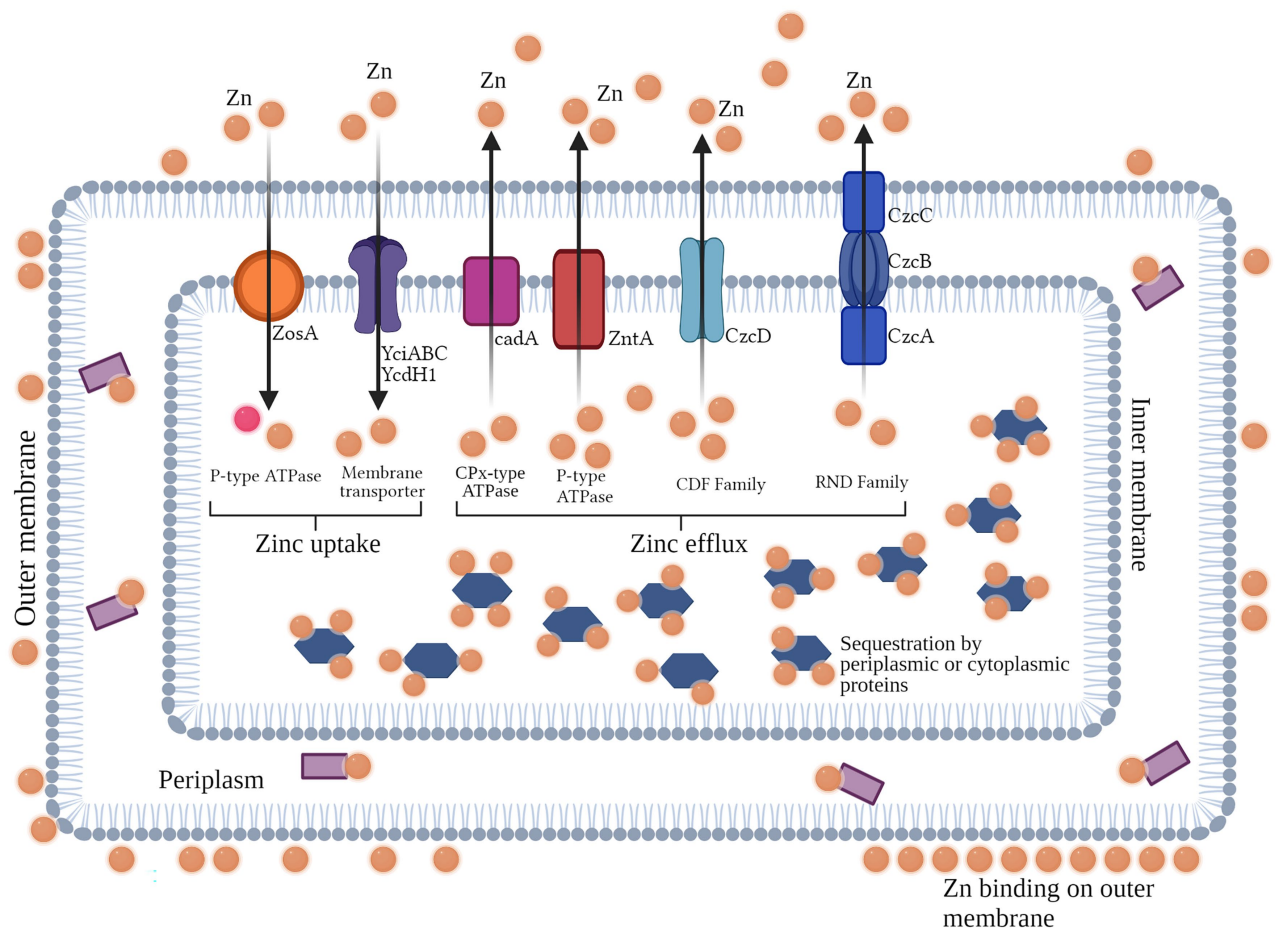
Overview of the general resistance mechanisms for Zn found in Gram-negative and -positive bacteria (Created with BioRender). In Gram negative and positive bacteria, general resistance mechanisms aid in efflux, sequestration, chelation, and metal uptake for their survival in toxic or scarce conditions. For the metal Zn, uptake in Gram positive bacteria from the outer environment is facilitated by P-type ATPase (ZosA), and Zn membrane transporters. For efflux of Zn ions out of the cell, CDF and RND efflux pumps (Czc determinants found in both Gram positive and negative bacteria), as well as CPx-type (CadA in Gram positive bacteria) and P-type ATPases (ZntA in both Gram positive and negative bacteria) are majorly involved. Moreover, intracellular or periplasmic proteins can bind to Zn inside the cell.

Intracellular concentrations of Zn are regulated through its homeostasis, where the use of a Zn regulator enables cells to regulate the transcriptional expression of Zn transporters which facilitate its import and export to and from the cell. This mechanism employed by cells is driven by conditions of Zn toxicity (efflux) and starvation (uptake) across the bacterial cell membrane (Capdevila et al., 2016). In bacteria, general resistance mechanisms pertain to both Gram-negative and -positive bacteria, with many agents overlapping in many species for a particular metal. These general mechanisms can aid in metal resistance through various ways, such as efflux, sequestration, chelation, and uptake. For Zn, several of these mechanisms are widely reported in many bacterial species. The intracellular regulation (uptake and efflux) of Zn^2+^ ions across the cell membrane is mediated by Zur/SlyA/MarR family and the MerR (ZntR) and ArsR/SmtB families, respectively (Mikhaylina et al., 2018). Zur is a member of the Fur family, which represses znuABC (an ABC transporter which facilitates Zn^2+^ ions uptake) under normal concentrations of Zn^2+^ in the cell (Hantke, 2001). However, in conditions of Zn^2+^ scarcity, Zur reverses its functions and induces the transporter to uptake Zn^2+^ from the outer environment. Moreover, it activates the transcription of a P-type ATPase which mediates the efflux of Zn^2+^ out of the cell under conditions of its toxicity (Capdevila et al., 2016). In *Streptococcus pneumoniae*, CzcD, a member of the CDF family, acts as major resistance determinant for Zn (Figure 2; Suryawati, 2018).

### Mechanisms of Zinc Uptake in Bacteria

In order to control Zn homeostasis, bacterial cells are able to enable the import and export of Zn ions *via* Zn uptake systems. In most bacterial species, two highly specified uptake systems are found to act in conditions of extreme toxicity (low-affinity uptake system) and extreme scarcity (high-affinity uptake systems), respectively, both of which are used in different conditions. In moderate conditions inside the cell, low-affinity uptake transporters are used to maintain levels of Zn (Hantke, 2001). The ABC family, comprised of three regulatory proteins (ZnuA, ZnuB, and ZnuC) is attributable to high-affinity uptake of Zn in the cell (Patzer and Hantke, 1999) and is a member of the ATP-binding cassette transporter family of the ZnuABC and AdcABC/ZitSPQ type. ZnuA is a soluble protein which resides in the outer membrane of the bacterial cell, whereas ZnuB is the protein which is found in the inner membrane and interacts with ZnuA. The third protein, ZnuC, regulates the breakdown of ATP, thus contributing to the uptake and efflux of the metal. Reports suggest that the deletion of ZnuA, ZnuB, and/or ZnuC or *znuABC* in different bacterial species can result in the decreased uptake of Zn (Porcheron et al., 2013). Though much is known about this high-affinity uptake system, very little is reported about the other uptake system (Chandra et al., 2007). In *Bacillus subtilis*, the uptake of Zn^2+^ is regulated majorly by Zur family, but another uptake system known as ZosA (which is a P-type ATPase) was also reported to be active in conditions of oxidative stress (Suryawati, 2018; Figure 2; Table 2).

**Table 2:** Genes involved in Zn resistance in Gram-positive and Gram-negative bacterial species.

Gene	Toxic ion(s)	Bacteria
*czcA*, *czcB*, and *czcC*	Zn, Cd, Co	*Ralstonia eutropha*
*czrCBA*	Zn, Cd	*Pseudomonas aeruginosa*
*zntA*	Zn, Co	*Staphylococcus aureus, Escherichia coli, Ralstonia metallidurans*
*smtAB*	Zn, Cd	*Synechococcus* sp.
*ycdH* operon *yciC* operon	Zn	*Bacillus subtilis*
*cadCA, cadB*	Zn, Cd	*Staphylococcus aureus*

### Mechanisms of Zinc Efflux in Bacteria

In conditions of Zn toxicity, the expression of efflux systems in bacterial cells aids in the prevention of overabundance of Zn (Guilhen et al., 2013). In bacteria, three families of exporters; cation diffusion facilitator (CDF), Resistance nodulation division (RND) efflux pumps, and P-type ATPases are majorly reported to be involved in metal export out of bacterial cells (Kolaj-Robin et al., 2015). Among these families, the CDF family is one of the most commonly found protein families in living beings. In *Escherichia coli*, ZitB and YiiP proteins, members of the CDF family, are transporters which regulate the efflux of Zn^2+^ ions *via* using the energy generated through the uptake of H^+^ ions (Porcheron et al., 2013). Another member of the CDF family, ZntA, reported to mediate resistance against Zn^2+^ ions in *Staphylococcus aureus*, is a well-known Zn transporter (Singh et al., 1999). In *Caulobacter crescentus*, the czrCBA system is the major efflux facilitator for Cd^2+^ and Zn^2+^ ions (Valencia et al., 2013). The Czc determinant confers resistance *via* efflux against various metals, such as Co^2+^, Zn^2+^, and Cd^2+^ ions in *Alcaligenes eutrophus* (Nies et al., 1989). This efflux system is a cation/proton anti-porter system which enables the efflux of cations from the cell, and consists of three structural genes (*czcABC*) which in turn encode three regulatory proteins of the efflux pump CzcA, CzcB, and CzcD (Nies, 1992). Moreover, efflux of Zn^2+^ ions in *B. subtilis* is mediated by CadA, which is a CPx-type ATPase efflux system (Gaballa et al., 2002). P-type ATPase family is reported to be found in both eukaryotes and bacteria, though they are generally well-defined in the latter. These transporters are regulated either by MerR/ZntR or ArsR/SmtB family members, which are often found to be working in close association with other metal transport proteins (Hantke, 2001). P-type ATPases regulate the transportation of metal ions across the bacterial cell membrane by utilizing the energy of ATP hydrolysis (Apell, 2004). A good example of P-type ATPase is ZntA in *E. coli*, which is regulated by MerR-like regulator *via* the attachment of apo-ZntR dimer to the promotor region of zntA, repressing its transcription (Porcheron et al., 2013). This phenomenon takes place when intracellular Zn concentration exceed sub-toxicity levels (Khan et al., 2002). The RND family is majorly found in Gram-negative bacteria which is involved in the active efflux of antibiotics and other therapeutic agents. These systems have large periplasmic domains and in many bacterial species are known to form complexes with extracellular channels and adaptor proteins (Nikaido and Takatsuka, 2009). In *C. crescentus*, CzrCBA is known to export cadmium and Zn ions from cytoplasm of cells (Valencia et al., 2013). The export of Zn and other metal ions in *Ralstonia metallidurans* is facilitated by CzcABC system, which is regulated by CzcS/CzcR two-component regulatory system (Anton et al., 1999; Hantke, 2001; Blencowe and Morby, 2003; Blindauer, 2015; Suryawati, 2018; Figure 2).

### Zinc Bioremediation by Bacteria

The bioremediation of Zn from various environments has been reported in various studies over the years. *Brevibacterium* sp. was effective in removing Zn from polluted environments, even in concentrations as low as 0.1 mm (Taniguchi et al., 2000). The biosorption potential of *Delftia tsuruhatensis* for Zn and lead (Pb) was also reported in a study (Bautista-Hernandez et al., 2012). Bacterial consortia of several species, such as *Alcaligenes faecalis, Staphylococcus aureus, Streptococcus lactis, Micrococcus luteus,* and *Enterobacter aerogenes,* was reported to remove various heavy metals, such as copper (Cu), cadmium (Cd), Pb, and Zn from wastewaters (Silambarasan and Abraham, 2013). *Pseudomonas putida*, another Gram-negative bacterium, was reported to demonstrate highest ability of Zn removal among other isolates in a study, whereas *Bacillus subtilis* was the most effective among Gram-positive bacteria, respectively (Kamika and Momba, 2013). *Rhodobacter capsulatus* was reported to efficiently remove Zn from polluted environments with the help of its wild-type strain and an enclosed plasmid which conferred resistance (Magnin et al., 2014). *Bacillus licheniformis* and *Salmonella typhi* were reported to remove more than 90% of Zn from contaminated samples, whereas *Pseudomonas fluorescens* and *E. coli* were effective in removing more than 96 and 93% of Zn, respectively (Basha and Rajaganesh, 2014). *P. fluorescens* and *P. putida* were also observed to be good bioremediation agents for many heavy metals, including Zn (Upadhyay and Srivastava, 2014; Ahmady-Asbchin et al., 2015; Naz et al., 2016). In a similar study, several bacterial species, such as *P. aeruginosa*, *S. aureus*, *E. coli, Proteus vulgaris,* and *Klebsiella pneumoniae,* were evaluated for their heavy metal removal ability, where their mixed consortium was able to remediate more than 90% of Zn. Among pure isolates, *P. aeruginosa* was effective in removing the largest quantity of Zn from contaminated medium (53.9%), as well as other heavy metals (Oaikhena et al., 2016). Among Gram-positive bacteria, *Staphylococcus epidermidis* was also reported to be effective in removing Zn (<80%) as well as other heavy metals from contaminated water (Nwagwu et al., 2017). *Streptomyces* sp. also demonstrated to remove Zn under controlled conditions (Sedlakova-Kadukova et al., 2019). Another study revealed *Serratia* sp. to be effective in tolerating high concentrations of Zn. High values of biosorption (more than 90%) were observed under Zn stress, which also aided in growth augmentation of plant roots, shoots, and chlorophyll content (Kour et al., 2019). Furthermore, the bioremediation of Zn by various *Bacillus* sp. from soil and wastewaters has been well-reported in various studies over the years (Joo et al., 2010; Singh and Chopra, 2014; Wierzba, 2015; Huang et al., 2020; Table 3). A recent study also sought out to investigate Zn removal efficacy of *Sporosarcina pasteurii* from contaminated soils, where urease-producing isolates and *S. pasteurii* had removal ability of more than 70%, and L-asparaginase-producing isolate had the ability to remove more than 90% Zn in solution (Ghorbanzadeh et al., 2022).

**Table 3:** Zn biosorption and metal uptake ability of various Gram-positive and Gram-negative bacterial species, as reported in different studies.

Bacterial strains	Initial metal concentration	Metal uptake	References
*Bacillus firmus*	100 mg/l	61.8%	Salehizadeh and Shojaosadati, 2003
*B. subtilis* D_215_	100 mg/l	63.73%	Sabae et al., 2006
*Bacillus jeotgali*	75 mg/l	30%	Green-Ruiz et al., 2008
*B. cereus*	0–200 mg/l	66.6 mg/g	Joo et al., 2010
*Pseudomonas* sp.	1 mm	49.8%	Kumaran et al., 2011
*Delftia tsuruhatensis*	–	0.207 mmoL/g	Bautista-Hernández et al., 2012
*Pseudomonas* sp. *SN7* *Pseudomonas* sp. *SN28* *Pseudomonas* sp. *SN30*	1.6 mM	29 mg/g 25 mg/g 26 mg/g	Ahemad and Malik, 2012
*Geobacillus thermodenitrificans* *Geobacillus thermocatenulatus.*	0.5 g/l	18 mg/l 24 mg/l	Babák et al., 2012
*Exiguobacterium* sp. ZM-2	25–200 mg/l	78.2%	Alam and Ahmad, 2013
*B. licheniformis*	0.1 mg/l	53%	Kamika and Momba, 2013
*B. licheniformis* *Salmonella typhi* *P. fluorescens* *Escherichia coli*	–	96.14% 91.78% 96.14% 93.27%	Basha and Rajaganesh, 2014
*Alcaligenes faecalis* *Staphylococcus aureus* *Streptococcus lactis* *Micrococcus luteus* *Enterobacter aerogenes*	–	39% 41% 38% 41% 67%	Silambarasan and Abraham, 2013
*Bacillus* sp. (KF710041) *B. subtilis* (KF710042)	–	73.29% 78.15%	Singh and Chopra, 2014
*Rhodobacter capsulatus*	10 mg/l	164 mg/g	Magnin et al., 2014
*B. subtilis*	178 mg/l	49.7 mg/l	Wierzba, 2015
*P. aeruginosa*	100 mg/l	46.1 mg/g	Ahmady-Asbchin et al., 2015
*P. aeruginosa* *S. aureus* *Escherichia coli* *Proteus vulgaris* *Klebsiella pneumoniae*	7 mg/l	53.9% 90.1% (Mix culture)	Oaikhena et al., 2016
*B. megaterium* EMCC 1013 *Rhizobium leguminosarum* EMCC1130	1 mg/ml	88% 85%	El-barbary and El-badry, 2018
*Serratia* sp.	1,000 mg/kg	< 90%	Kour et al., 2019
*Streptomyces* K11 strain	–	36%	Sedlakova-Kadukova et al., 2019
*Stenotrophomonas maltophilia* XZN4	20 g/l	91.6%	Huang et al., 2020
*Oceanobacillus profundus*	2 mg/ml	54%	Mwandira et al., 2020
*Sporosarcina pasteurii* Urease-producing isolate L-asparaginase-producing isolate	4 mmol/l 8 mmol/l	70.36% 71.46% 97.32%	Ghorbanzadeh et al., 2022

## Conclusion

Zn is the one of the major elements (23rd) found in the Earth’s crust, with an average concentration of approximately 78 mg/kg. After Fe, Al, and Cu, it is the major metal used in the world, in terms of tonnage. Zn is regarded as a biologically essential element for living beings. It is reported to be imperatively required for the normal functioning of major enzymes and proteins. It is also reported to be effective in combating viral infections and strengthening the immune system against them, which involves the inhibition of the entry, fusion, replication, translation, and emission of virus particles into and out of the host cells. However, excess levels of Zn can act as toxic, which can induce conditions of acute toxicity for living beings. For its mitigation from the environment, bioremediation is a comparatively cheaper, more efficient, and effective method employed for the removal of heavy metals from various environments, like effluents and wastewaters. In bacteria, general resistance mechanisms (such as efflux, uptake, sequestration, and chelation) are found in Gram-negative and -positive bacteria which aid in the removal of heavy metals, such as Zn, from polluted environments. However, these resistance mechanisms need to be investigated on a molecular level so that these species could be manipulated for industrial applications, as one of the drawbacks of bioremediation is its inability to be reproduced from lab scale to large scale. This review focuses on the uses, toxicity, and bioremediation of Zn with special insight into its chemical and physical properties as well as its role in nutrition and immunity. Further innovative aspects of Zn remediation and toxicity can aid in the elucidation of resistance mechanisms, as well as Zn bioavailability.

## Author Contributions

All authors contributed equally in this manuscript. All authors read and approved the submitted version.

## Conflict of Interest

The authors declare that the research was conducted in the absence of any commercial or financial relationships that could be construed as a potential conflict of interest.

## Publisher’s Note

All claims expressed in this article are solely those of the authors and do not necessarily represent those of their affiliated organizations, or those of the publisher, the editors and the reviewers. Any product that may be evaluated in this article, or claim that may be made by its manufacturer, is not guaranteed or endorsed by the publisher.

## Abbreviations

ATP: Adenosine 5′-triphosphate
CDF: Cation diffusion facilitator
DNA: Deoxyribonucleic acid
IFN-γ: Interferon gamma
IL-1α: Interleukin 1 alpha
IL-1β: Interleukin 1 beta
IL-2: Interleukin-2
IL-6: Interleukin-6
NADPH: Reduced nicotinamide adenine dinucleotide phosphate
NF-κB: Nuclear factor kappa B
NK: Natural killer
RNA: Ribonucleic acid
RND: Resistance nodulation division
ROS: Reactive oxygen species
SARS: Severe acute respiratory syndrome
TLR: Toll-like receptor
TNFR: Tumor necrosis factor receptors
TNF-α: Tumor necrosis factor α
