# Corrigendum: Zinc essentiality, toxicity, and its bacterial bioremediation: A comprehensive insight

**DOI:** 10.3389/fmicb.2022.1133733

**Published:** 2023-01-17

**Authors:** Sarfraz Hussain, Maryam Khan, Taha Majid Mahmood Sheikh, Muhammad Zahid Mumtaz, Talha Ali Chohan, Saba Shamim, Yuhong Liu

**Affiliations:** ^1^Key Laboratory of Integrated Regulation and Resource Development on Shallow Lakes of Ministry of Education, College of Environment, Hohai University, Nanjing, China; ^2^Institute of Molecular Biology and Biotechnology, The University of Lahore, Lahore, Pakistan; ^3^Institute of Plant Protection, Jiangsu Academy of Agriculture Sciences, Nanjing, China

**Keywords:** zinc, bioremediation, pollution, wastewater, heavy metal, toxicity

In the published article, there was an error in the **Acknowledgments** section, page 10, paragraph 1. The correct statement appears below.

